# Non-detection of honeybee hive contamination following *Vespula* wasp baiting with protein containing fipronil

**DOI:** 10.1371/journal.pone.0206385

**Published:** 2018-10-29

**Authors:** Eric D. Edwards, Ethan F. Woolly, Rose M. McLellan, Robert A. Keyzers

**Affiliations:** 1 Department of Conservation, Wellington, New Zealand; 2 School of Chemical and Physical Sciences, Victoria University of Wellington, Wellington, New Zealand; 3 Centre for Biodiversity and Restoration Ecology, Victoria University of Wellington, Wellington, New Zealand; Institut Sophia Agrobiotech, FRANCE

## Abstract

Introduced wasps (*Vespula germanica* and *V*. *vulgaris*) are costly invertebrate pests in New Zealand, with large impacts on the local ecology and economy. Wasps eat honeybees (*Apis mellifera*), which has potentially devastating effects on hive health, as well as agricultural and horticultural industries. Vespex bait, which contains fipronil in a proteinaceous carrier, has recently been introduced for wasp control. In over a decade of reported trials, honeybees have never been observed foraging on Vespex, likely because the bait contains no sugars to serve as a bee food source. However, the potential for the control agent fipronil to enter beehives has not been tested. Therefore, here, we investigated this using a liquid chromatography–mass spectrometry assay of fipronil and two of its environmental breakdown and metabolic derivatives, fipronil desulfinyl and fipronil sulfone. We did not detect fipronil in any of the worker bee, bee larva, honey or pollen samples (n = 120 per product) collected from 30 hives over a 2-year period. Furthermore, although we detected fipronil desulfinyl in one honeybee sample, this is thought to have originated from a single individual, representing a rare occurrence of intoxication, and there was no evidence that Vespex was the toxicant source. There was also no evidence of trophallactic transfer of fipronil or its derivatives in any of the hives sampled. Previous studies have reported the impairment of individual bee performance at fipronil doses similar to the detection limit of our study. However, our results provide confidence that if undetectable intoxication was occurring, it would involve an acute exposure for those few individuals affected, with minimal impairment to colonies. Therefore, we conclude that the use of Vespex in the vicinity of honeybees does not result in significant hive uptake while effectively reducing wasp pressure on honeybee colonies.

## Introduction

Unintentional, non-target exposure during the broad-scale use of insecticides has been recognised as a contributing factor to the loss of honeybees (*Apis mellifera* L. 1758) worldwide [1–8]. However, the use of chemical insecticides remains the most effective method for suppressing many insect pests. Therefore, it is important that non-target effects, particularly on native or economically valuable species, are minimised during insect pest interventions.

Two exotic species of wasps have been accidentally introduced to New Zealand: *Vespula germanica* (Fabricius, 1793) in 1945 and *V*. *vulgaris* L., 1758 in the late 1970s [9–10]. Both species are now distributed throughout New Zealand, but *V*. *vulgaris* is most abundant. Their pest status is derived from the harm they bring to native species and ecosystems [11–15], as well as the local economy [16]. *Vespula* wasp attacks on honeybees have been widely reported [14, 17–20], and it has been conservatively estimated that New Zealand beekeepers lose NZ$8.8M per year as a result of wasps taking honey and bees from hives, bees defending their hives instead of foraging, and the protection of hives from wasp attack, which includes the use of insecticides [16].

A new method for suppressing wasps over a wide area recently became available in New Zealand [21]. This involves placing bait stations on trees in areas where wasps are active and filling them once per year with a protein wasp bait containing 1 g/kg fipronil active ingredient (traded as Vespex). Since this bait does not contain any sugars, it is improbable that honeybees will visit. However, since hive safety is of paramount importance in any insecticide-based control strategy, the dispersal and potential uptake of fipronil into hives needs to be assessed to ensure that it is safe to use in the field.

### Potential for honeybee exposure

One factor that limits fipronil exposure for invertebrates other than *Vespula* wasps is the lack of sugars in the bait matrix, which consists of a fresh protein paste. When wasps are abundant and are shown to be seeking protein [21], 20–30 g of this paste is placed in bait wells that are inserted into weatherproof bait stations mounted on trees approximately 1.5 m above the ground, making them accessible to winged insects. The potential period of exposure to the bait is brief, as any residual paste dries to a solid within 3 days and is removed within 8 days of initial placement, and treatment is not repeated until the following year.

Worker wasps carrying bait have a limited ability to digest protein and so fly to their nest, where the larvae are able to share any digested food, spreading fipronil active ingredient throughout the wasp colony. These colonies are usually found in dry soils, and any dead wasp larvae, pupae and adults decay *in situ* and can be scavenged by rodents. Any fipronil residues would therefore be concentrated at wasp nest sites and become bound to organic materials. Bait stations can be located 50 m apart but are usually at a lower density. Based on the recommended dosing regimen, the maximum amount of fipronil and its metabolites that would enter the environment per 10,000 m^2^ per year is 0.12 g, which would be achieved by wasps taking all of the bait to their nests within 2–3 days. However, this amount is more typically <0.02 g per 10,000 m^2^.

There is no obvious pathway by which Vespex would have lethal or sub-lethal effects on honeybees. However, there are four theoretical pathways for honeybee exposure to fipronil. First, honeybee workers occasionally enter wasp bait stations, which would bring them in dermal contact with the bait or oral contact while seeking mineral salts [22–23]. Second, moribund intoxicated wasps may enter bee hives. Third, residues of fipronil and its environmental degradation and metabolic products may be taken up systemically by plants at the wasp nest site, which may be subsequently visited by bees [24–25], although it seems unlikely that this would occur at a scale that could harm bee colonies and so this pathway was not addressed by this study. Finally, insecticide containing fipronil active ingredient is sprayed onto onion crops (*Allium* spp.: Amaryllidaceae) and stock food brassicas (*Brassica* spp.: Brassicaceae) in New Zealand, which could represent an alternative environmental source [26–27].

### Detecting honeybee colony impacts

Many studies that have assessed the impacts of fipronil and other insecticides on honeybee colonies have been laboratory based and have only considered the lethal and sub-lethal effects on individual bees [28–30]. Therefore, it can be challenging to transfer the findings to field situations of normal apicultural operations. Where a hive fails or has numerous moribund and dead bees that test positive for a toxic agent (or its metabolites), this can be linked to inappropriate insecticide use. However, hive failure can often occur in the presence of multiple environmental stress factors, with no obvious single cause [8, 31]. In many countries, there is great concern that the additive effects of environmental stress factors, including sub-lethal insecticide exposure, may cause widespread hive losses or reductions in pollination services from hives [1, 8, 32–34], and attempts to determine the adverse effects of sub-lethal insecticide exposure have linked the detection of trace amounts of insecticide in the hive with laboratory research results for behavioural and physiological effects [8, 33, 35].

A small number of studies have shown that individual worker bees fail to perform at doses of fipronil and its metabolites that are below the limit of detection (LOD) of contemporary assay methods, particularly in the case of thorax dermal contact with solutions [29, 30, 36, 37]. Some studies have also attempted to trial a limited period of sub-lethal exposure to fipronil in the environment and then measure hive performance and metrics of hive health in the following months [38–40]. However, a range of confounding factors could affect these results, particularly where hive health and bee performance monitoring occurs weeks or months later [8].

In New Zealand, the potential for bees to be exposed to fipronil when the wasp bait station method is used is acute (via bait or intoxicated wasps; from dermal or oral contact; and within 1–3 days) rather than chronic repeated doses over a week or more from contaminated plants. Consequently, it would be challenging to design a series of treatments and controls that would isolate a potentially enduring hive impact (or lack thereof) from a brief exposure to an undetectably low amount of fipronil [8].

In this study, we chose to seek direct evidence of any colony-wide exposure by developing a test for fipronil and its derivatives that was sufficiently sensitive to detect sub-lethal quantities of up to one-tenth of a conservatively reported median lethal dose (LD_50_) and then testing numerous samples from multiple hives situated at sites where wasps were baited using Vespex with fipronil as the active ingredient. We limited our data collection to the trace detection of fipronil and its derivatives, and sampled worker bees, bee larvae and other bee products. However, we regarded nurse and forager bees to be one of the best indicators of colony intoxication at the time of sampling as they are centrally involved in both the distribution and redistribution of all foods [41].

Chromatographic methods have been proven to be the premier approach for the qualitative and quantitative analysis of metabolites, herbicides and insecticides [42–44]. Therefore, we adapted a method for processing bees and a variety of bee products that utilised a QuEChERS (Quick, Easy, Cheap, Effective, Rugged, Safe) protocol [44] followed by liquid chromatography–tandem mass spectrometry (LC-MS/MS) analysis with a quadrupole-time of flight (Q-TOF) mass spectrometer. We initially validated the use of this method for the detection of sub-lethal levels of fipronil and two of its environmental and metabolic breakdown derivatives, fipronil desulfinyl and fipronil sulfone. We then used it to analyse a total of 480 samples of worker bees, bee larvae, honey and pollen (n = 120 per product; Table 1) over 2 consecutive years to assess the uptake of fipronil following its *in situ* application for wasp control.

**Table 1:** Timeline of honeybee hive sampling and wasp treatment.

Site	Activity	Date	Number of samples^d^
Big Bush (season 1)	Hives sampled (prior^a^)	20 Feb 2015	80
	Wasps treated^b^	20–24 Feb 2015	
	Hives sampled (after^c^)	27 Feb 2015	80
Big Bush (season 2)	Hives sampled (prior)	3 Feb 2016	80
	Wasps treated	6–10 Feb 2016	
	Hives sampled (after)	12 Feb 2016	80
Pelorus Bridge	Hives sampled (prior)	4 Feb 2016	80
	Wasps treated	22–26 Feb 2016	
	Hives sampled (after)	26 Feb 2016	80
			TOTAL 480 assays

^a^ Hives were sampled prior to wasp treatment in the hive environment.

^b^ Wasp bait was placed in bait stations in the environment of the bee hives.

^c^ Hives were sampled after wasp treatment in the hive environment. Hives located in associated non-treatment areas were also sampled on the same date as in the treatment areas.

^d^ Four products (honeybees, bee larvae, honey and pollen) were sampled in 10 hives in each of the treatment and non-treatment areas. In 2015, hive scrapings were used in place of fresh pollen.

We acknowledge that a lack of detection would not mean that individual bees were not harmed and that whole colonies could be harmed by sub-lethal quantities that remain below the detection limit for all samples. However, we considered this unlikely given the brief period of protein bait exposure (where the bait has a high concentration, 1 mg/g) and the study design used.

## Materials and methods

Permission for field investigative work with pest wasps and honeybees was granted by New Zealand Department of Conservation to EDE as an employee of Department.

### Study sites

We sampled honeybee hives in two regions in the South Island of New Zealand that were >75 km apart: Big Bush Conservation Area (BB; BB; 41°17′51″S, 173°34′46″E WGS 84), which includes extensive native beech (*Fuscospora* spp.) forest and some tall Myrtaceae-dominated shrub-land; and Pelorus Bridge Scenic Reserve (PB; 41°47′08″S, 172°49′09″E WGS 84), which includes beech and hardwood forest. Both sites include agricultural grassland and herb-field areas. These sites were selected because beekeepers place bee hives in beech forest areas on a seasonal basis to allow the bees to collect honeydew, which is produced on beech trees by native scale insects (*Ultracoelostoma* spp.: Coelostomidiidae). However, pest wasps (*Vespula* spp.) also reach high abundances on the same carbohydrate resource [11].

At BB, a group of 10 hives was surrounded by 9 wasp bait stations placed on trees 1.5 m above the ground at a distance of 20–120 m from the hives, while a second group of 10 non-treatment control hives was placed 1,900 m from the nearest wasp bait station. At PB, a group of 10 hives was placed at the boundary between forest and pasture, and 325 wasp bait stations were placed at 50-m intervals along transects that were 100 m apart along the same forest boundary and inside the forest but not in the pasture, with a distance of 20 m between the hives and the nearest bait stations. These wasp bait stations were distributed over 2.17 km^2^ of forest, giving a lower density but broader area of coverage than at BB. A second group of 10 non-treatment hives was located 4,500 m from the nearest wasp bait station.

### Wasp control

The experiment was repeated over two seasons at BB (February 2015 and 2016) but was only carried out in one season at PB (February 2016) (Table 1). Starting in February, we examined wasp activity on protein foods following the Vespex manufacturer’s instructions. We baited 20 trays placed 5 m apart with approximately 15 g non-toxic fish bait and left them for 1 hour, following which we conducted an instantaneous wasp count. We planned a bait station wasp control operation when the mean wasp count was ≥1 wasp per tray over 20 trays. As soon as at least 3 days of fine weather was forecast (no rain and locally warm air temperatures), bait stations were filled with 20–30 g Vespex wasp bait (a green protein paste without sugars but containing 1 g/kg fipronil). Since any residual bait paste dries to a hard solid within 3 days, any bait remaining in the bait stations was removed after 5 days and according to standard practice was not replaced.

### Honeybee hive sampling

To test whether fipronil was already present in the environment, sampling was undertaken prior to wasp control and in nearby, non-treatment hives. Groups of 10 hives were acclimated to each site at least 10 days prior to the first sampling event. During sampling, a professional beekeeper inspected the queen honeybee and collected a minimum of 20 g of worker bees, 2 g of bee larvae from uncapped cells, 2 g of honey from uncapped cells and pollen. In 2015, scrapings of pollen, propolis and wax were collected from each hive, while in 2016, pollen traps were installed in the base of the hives 2–4 days prior to sampling. Tools were cleaned or replaced prior to sampling each hive.

The four products were assayed separately, giving a total of 160 samples per season per area (four hive products from 10 treatment and 10 non-treatment hives across two sampling periods [before and after treatment]). Sampling was carried out twice at BB and once at PB (Table 1), giving a total of 480 samples across the entire study (n = 120 per product). The samples were couriered overnight on ice to an analytical laboratory (School of Chemical and Physical Sciences, Victoria University of Wellington, New Zealand) and stored at −20°C until analysis, which was carried out within 4 months of receipt. Few studies have examined the metabolic fate of fipronil in insect species, but results indicate that *in vivo* metabolism primarily converts this toxicant into the sulfone form and that most fipronil is recovered in the parent form 24 hours after exposure [45]. In addition, both the desulfinyl and sulfone forms can be produced photochemically [46–47]. Therefore, to maximise the likelihood of detecting any fipronil intoxication and transfer within the hive, post-treatment samples were taken within 1–3 days of wasp treatment.

### Sample preparation

All solvents (acetonitrile and hexane) that were used for sample preparation and analysis were of analytical grade and were purchased from Fisher Scientific (Suwanee, GA, USA). Water was purified with an Arium 611UV system (Sartorius AG, Germany). The QuEChERS kits, including ceramic homogenisers, extraction and purification salts, and primary secondary amine (PSA), were obtained from Agilent Technologies, Inc. (Folsom, CA, USA). Fipronil (98% purity) was purchased from AK Scientific (Union City, CA, USA), while its two environmental and metabolic derivatives (fipronil sulfone and fipronil desulfinyl) and the internal standard (IS; thiacloprid), which were of 99.9% purity, were purchased from Sigma-Aldrich (St. Louis, MO, USA). Mixed standard solutions were prepared in acetonitrile.

Sample analysis for the presence of fipronil and its derivatives followed the methods of Kasiotis et al. [43] and Paradis et al. [44]. Briefly, sample preparation was optimised for a mass of 1 g of worker bees, bee larvae, honey or pollen. The average weight of an individual honeybee was estimated by weighing groups of 50 bees to standardize the sample sizes used. Each sample was transferred to a 15-mL Falcon tube and mixed with ceramic homogenisers and extraction salts (0.5 g of MgSO_4_, 0.2 g of NaOAc and 0.2 g of PSA) in deionised water (3 mL) and shaken vigorously for 2 minutes. The IS (1 mL, 400 ng/mL in acetonitrile) was then added along with an additional 6 mL of acetonitrile and 2 mL of hexane. The sample dispersion was centrifuged at 4,000 × *g* for 5 minutes at 15°C, following which the supernatant (7 mL) was recovered, transferred to a new Falcon tube containing purification salts and a stationary phase (1.2 g of MgSO_4_, 0.4 g of PSA and 0.4 g of C18EC) and vortexed for 2 minutes. This was then filtered through cotton wool into a test tube and the retentate was washed with acetonitrile (3 mL). The solvent was evaporated to dryness and then reconstituted in 3:2 acetonitrile/water (1 mL), which was filtered through a 0.45-μm PTFE disk into 2-mL LC-MS vials for analysis. To establish calibration curves (1–100 ng/mL), commercially available blank honey was analysed in which no fipronil or fipronil derivatives were detected, and this honey was then spiked with pesticide standard solutions and mixed with a vortex mixer to ensure homogenous distribution. All samples and calibration standards were analysed in triplicate, and all analyses were performed blind in the laboratory, with analysts unaware of the source of the samples.

There were some minor differences in sample preparation between 2015 and 2016. In 2015, fipronil desulfinyl was not available as a standard and so was not tested for. In addition, no IS was used in 2015, with sample recoveries instead being monitored by using blank honey spiked with 1 mL of a 200 ng/mL fipronil and fipronil sulfone calibration solution for every 24^th^ sample.

### LC-MS/MS analysis

Liquid chromatography analysis was carried out using an Agilent Technologies (Palo Alto, CA, USA) 6530 Q-TOF LC-MS system equipped with a Kinetex C18 column (XB-C18; Phenomenex; 2.6 μm particles, 100Å, 50 × 2.10 mm). The chromatographic gradient was optimised for separation of fipronil and its two derivatives along with the IS (S1 Fig). Chromatographic separation was performed at 25°C with an injected volume of 1 μL. The mobile phase was a mixture of (A) water (5 mM ammonium formate) and (B) acetonitrile (0.1% formic acid) with a flow rate of 0.4 mL/min. The target compounds were separated with the following gradient: 50% (B) from 0 to 0.5 minutes; a linear gradient from 50% to 90% (B) from 0.5 to 60 minutes; 90% (B) from 6 to 7 minutes; a linear gradient from 90% to 50% (B) from 7 to 10 minutes; and 50% (B) from 10 to 12 minutes to return to the initial conditions. Ionisation was performed with an electrospray ionisation source (ESI) heated at 300°C in positive-ion mode for the IS and negative-ion mode for fipronil and its derivatives. The following ion source conditions were used: capillary voltage, 2.75 kV; gas flow, 9 L/min; nebuliser, 30 psi; nozzle voltage, 500 V; fragmentor voltage, 140 V; MS acquisition rate, 2 spectra/s; MS *m/z* range, 100–1100; MS/MS acquisition rate, 2 spectra/s; MS/MS *m/z* range, 50–500; collision-induced dissociation (CID) gas, N_2_; CID gas pressure, 16 psi; isolation width, *m/z* 4; collision energies, 17 or 20 eV; total analysis time, 10 minutes. Any pesticide signals that were detected were normalised to the IS intensity. Agilent Technology’s MassHunter Workstation software, including Qualitative Analysis and Quantitative Analysis (version B.06.00), was used to process the MS and MS/MS data.

### Method validation

The LC-MS/MS method [44] was evaluated using several criteria (linearity, recovery rates, repeatability and specificity; see S1 Table) prior to its implementation for real-world samples. Calibration was performed using authentic mixed standards at 11 different concentrations and was repeated using blank honey samples spiked with the pesticide standard solutions. The linearity of the calibration curves was evaluated by obtaining determination coefficients without forcing the line of best fit through zero. Recovery rates were calculated as the ratio between the measured area counts of the spiked and non-spiked samples. The repeatability, specificity, LOD (signal to noise ratio [SNR] 3:1) and limit of quantitation (LOQ; SNR 10:1) were determined in accordance with the International Conference on Harmonization (ICH) guidelines [48]. Fifty worker honeybees were weighed in triplicate to estimate the average weight per bee, to allow validation of the results relative to individual bees.

## Results

### Method validation

The samples of 50 worker honeybees weighed 6.143 g, 7.436 g and 6.589 g (mean = 6.723, standard error = 5.64%), providing an estimate of 0.134 g per bee or 7 honeybees per gram.

The calibration curves had very good linearity, with *r*^2^ values of >0.99 for the standards and >0.98 for the spiked samples (Table 2). The recovery rates for fipronil and its derivatives were relatively low, ranging between 35% and 44%, with relative standard deviations of <11% indicating a high repeatability of the measurements (S1 Table). Specificity was ensured through the use of a retention time (± 0.5 min) and quantifier:qualifier ion ratios (± 0.25–0.45) to avoid false identifications (Table 2). The LODs for fipronil and its two derivatives ranged between 1.1 and 1.8 ng/mL, corresponding to 0.16–0.26 ng/bee or 1.1–1.8 ng/g of bee product, while the LOQs fell between 1.8–2.0 ng/mL (0.25–0.29 ng/bee), which are within the sub-lethal levels needed to observe toxicological effects on bees and below the most conservative reported ingested LD_50_ of fipronil for honeybees (1 ng/bee; see S2 Table and [36]).

**Table 2:** Figures of merit for the LC–MS/MS analysis of fipronil and its derivatives.

Compound	Retention time (min)	Parent ion ([M-H]^−^)	Collision energy (eV)	Quant. ion	Qual. ion	Quant./qual. ratio	LOD (ng/mL)	LOQ (ng/mL)	*r*^2^
Fipronil	2.3	434.924	17	329.953	249.954	0.25	1.1	2.0	0.991
Fipronil desulfinyl	2.8	386.964	17	350.959	281.991	0.45	1.8	1.9	0.988
Fipronil sulfone	3.3	450.916	20	414.941	281.987	0.25	1.6	1.8	0.985

LC–MS/MS, liquid chromatography–tandem mass spectrometry; quant., quantification; qual., qualification; LOD, limit of detection; LOQ, limit of quantitation.

### Testing for the presence of fipronil and its derivatives in honeybee hives

None of the 160 samples (40 samples each of honey, worker bees, bee larvae and a mix of pollen, wax and propolis) that were examined in 2015 tested positive for fipronil above the LOD. Three honey samples that were collected prior to bait deployment did return trace levels (between the LOD and LOQ) of fipronil sulfone, however, although all of these failed to register above the LOD for any of the compounds of interest when freshly prepared samples were retested.

Among the 320 samples comprising honey, worker bees, bee larvae and pollen that were examined in 2016, only one field sample of worker bees from the wasp treatment area in BB returned a positive result for any of the compounds of interest. This sample tested positive for fipronil desulfinyl (6 ng/mL) at a level that was well above the LOQ and at the typical 24–48-hour LD_50_ for an individual bee for both oral and dermal routes of exposure (see S2 Table). However, two additional re-assays of bees from the same field sample yielded negative results for this compound, as well as for fipronil and fipronil sulfone.

## Discussion

For fipronil and indicator derivatives, a detection limit of 1.1–1.8 ng/mL converts to 0.16–0.26 ng/bee. This is close to one order of magnitude less than a conservatively reported LD_50_ for a worker bee (S1 Table). However, a range of adverse behavioural and physiological effects for individuals are reported at and below this level of intoxication [1,28,29,30,36,37,49].

We found no trace of fipronil above its detection limit in any of the worker bee, bee larva, honey or pollen samples collected from either study area across both seasons following Vespex bait treatment for wasp control (Table 2). Furthermore, although we detected 6 ng/mL of fipronil desulfinyl in one sample assay containing approximately seven worker bees that was collected following wasp treatment in BB, two further assays from the same bee sample did not detect any of this compound. This suggests that a single bee was likely responsible for the result in the original sample and that this was a rare event, as detections would have been expected in the follow-up assays (or indeed in assays from other co-located hives) if multiple bees were involved in the same sample. However, this level of fipronil desulfinyl is equivalent to the LD_50_ for a single bee following dermal contact or direct ingestion of fipronil (see S2 Table). To the best of our knowledge, no toxicological data have been published to date for fipronil desulfinyl, but a wide range of oral toxicity values have been cited in the literature for fipronil and its other common derivative, fipronil sulfone (see S2 Table and [36, 38, 43, 49–50]).

We acknowledge that honeybees carrying small amounts of fipronil or its derivatives may have been missed through the random selection of bees during both field and laboratory sampling coupled with the low extraction recoveries obtained. Our choice of laboratory sample size (1 g of honeybees) was based on the published protocol of Kasiotis et al. [43] but contrasts with the 5 g of honey used by Paradis et al. [44]. There are advantages and disadvantages to choosing both smaller and larger sample sizes, as smaller sample sizes will avoid the dilution of toxicant residues by any additional extracted biomass but may result in individual positive samples being missed, while larger sample sizes will sample a wider range of the population but may dilute individual insecticide levels. If the 6 ng/mL of fipronil desulfinyl that was detected originated from one individual honeybee, an increase in sample size to 5 g would have resulted in an 80% dilution in the amount detected to 1.2 ng, which is close to our LOD. In addition, the detection of fipronil desulfinyl in 1 out of 80 samples from two study sites is unlikely to have increased had the sample size been doubled but would have imposed significant constraints in terms of cost and time. Therefore, the protocol was optimised through the use of a sensitive method combined with a limited laboratory sample size. In addition, the risk of under-sampling due to the use of a smaller extraction mass can be offset by testing multiple hives (see Table 1), as carried out in this study.

As mentioned above, the finding that no other samples from the same hive or from 29 other hives in wasp treatment areas contained fipronil desulfinyl suggests that this result may have originated from a single honeybee. Assuming the complete photochemical degradation of fipronil to fipronil desulfinyl (see [47]), 6 ng of fipronil desulfinyl could have originated from approximately 10 μg of Vespex bait, which we posit could, for example, have been inadvertently transferred to a single honeybee via walking on or ingesting the protein bait. Alternatively, it is possible that this resulted from an alternative source of contamination, such as a nearby agricultural crop spraying event, as indicated by the pre-treatment result for fipronil sulfone (see below).

The potential exposure of honeybees to fipronil or its derivatives during wasp control will differ from that experienced when fipronil is used as an active ingredient in insecticides for crop seed treatment, crop sprays or ground treatment for termites [51]. In horticultural applications, fipronil insecticide is often applied either as a dust coated on seeds or in aqueous solutions as a spray [51]. The application of fipronil in Vespex differs from this in two ways. First, the active ingredient is present in fresh protein paste wasp baits that are housed in weatherproof trays at a density of no more than three trays per 10,000 m^2^ and often less than one tray per 10,000 m^2^. Second, residual wasp bait that is not taken by pest wasps becomes a dry solid within 3 days and is removed from the bait stations after 3–8 days without replacement. Consequently, the environmental exposure of bee colonies to the toxicant (or its environmental or metabolic breakdown products) and the period over which they could develop sub-lethal effects is shorter than indicated by published toxicity tests [36, 40, 52–53], although even brief periods of exposure can result in subtle sub-lethal effects with both immediate and delayed effects.

The pathways by which fipronil can be transferred from wasp bait to the bee hive are also limited. Exposure could result from the direct contact of worker bees with the bait, with the entry of intoxicated *Vespula* wasps being less likely; however, neither scenario has been observed by the authors or reported by beekeepers who have used Vespex. It should also be noted that worker bees appear to have a negligible role as vehicles of fipronil associated with wasp baiting as we found no evidence for the trophallactic transfer of fipronil or its derivatives.

Three honey samples that were obtained from both study areas before wasp baiting occurred returned trace amounts of fipronil sulfone but not fipronil. The retesting of each sample yielded negative results but we cannot exclude the possibility that trace levels of fipronil sulfone just below the LOD were present. Therefore, we consider that these results indicate the low-level presence of this toxicant. The source of these detections was not identified but several possible scenarios exist. First, cross contamination may have occurred in the laboratory. However, this seems extremely unlikely as mixed standards were used and only 3 out of 480 samples returned positive results for only one fipronil derivative. Long-term stability studies on the soil degradation of fipronil have shown that this toxicant can take almost a year to break down (t_1/2_ 111–350 days; [54]). Therefore, given that honeybees do not consume any protein-based foods except pollen, any detection of fipronil in pre-baiting samples will be from historical applications of fipronil in the vicinity of the target sites. Consequently, it is possible that contamination occurred during field sampling or from contaminated nectar sources that were associated with the use of foliar sprays containing fipronil as an active ingredient adjacent to our test sites and within the flight range of honeybees.

These positive results, together with the low LOD/LOQs obtained, demonstrate that the method used was capable of detecting fipronil and its derivatives in bee and bee-product samples–a series of otherwise negative results could bring into question the validity of the chosen method.

>90% reductions in the activity of wasps from the use of Vespex wasp bait is previously reported for similar sites [21]. Since the impacts of the exotic wasps *V*. *vulgaris* and *V*. *germanica* on bees and bee services have been estimated to cost the New Zealand economy over NZ$70M annually [16], our finding that Vespex was not detected in bee colonies is important. However, there is ongoing concern about the impacts of *Vespula* wasps globally [13, 14], including in Australia [55], Argentina [56–57] and the islands of Hawai’i [58–59], indicating that wide-scale studies on the impact of wasp baits on bee populations are required.

## Supporting information

S1 Fig Extracted ion chromatogram of fipronil (2.36 min), fipronil desulfinyl (2.80 min) and fipronil sulfone (3.31 min). (PDF)

S2 Fig High-resolution electrospray ionisation tandem mass spectrometry (HR-ESI-MS/MS) fragmentation of fipronil, fipronil desulfinyl and fipronil sulfone. (PDF)

S3 Fig Calibration curves for fipronil and its derivatives. Values are means ± standard errors.
(PDF)

S1 Table Calculations of figures of merit for fipronil and its derivatives. (PDF)

S2 Table Summary of published fipronil LD_50_ toxicity data for honeybees (*Apis mellifera*). (PDF)
