# Supplementary material for: Non-detection of honeybee hive contamination following Vespula wasp baiting with protein containing fipronil
Source: PLoS One. 2018 Oct 29;13(10):e0206385. doi: 10.1371/journal.pone.0206385 (PMC6205613; doi:10.1371/journal.pone.0206385)
Supplement: S1 Fig — (PDF) [file pone.0206385.s001.pdf]

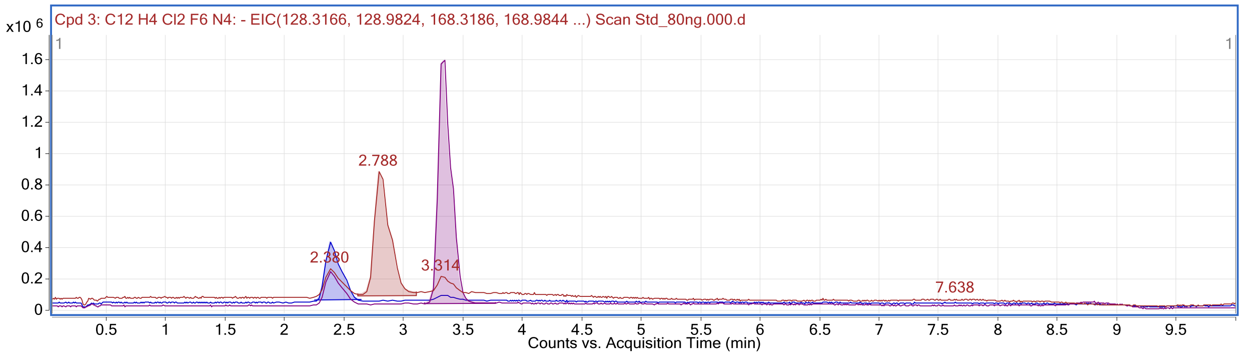


**S1 Fig. Extracted ion chromatogram of fipronil (2.36 min), fipronil desulfinyl (2.80 min) and fipronil sulfone (3.31 min).** Injection volumes were 1 μL.
