# Supplementary material for: Non-detection of honeybee hive contamination following Vespula wasp baiting with protein containing fipronil
Source: PLoS One. 2018 Oct 29;13(10):e0206385. doi: 10.1371/journal.pone.0206385 (PMC6205613; doi:10.1371/journal.pone.0206385)
Supplement: S2 Fig — (PDF) [file pone.0206385.s002.pdf]

Fipronil (17 eV)

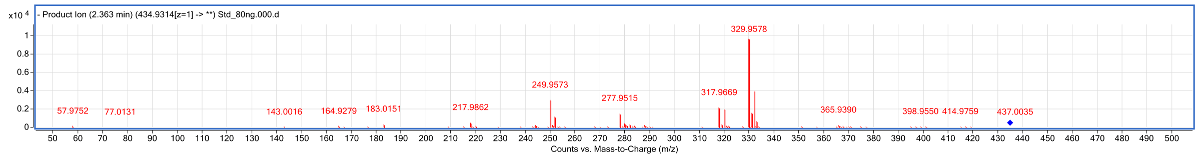

Fipronil desulfinyl (17 eV)

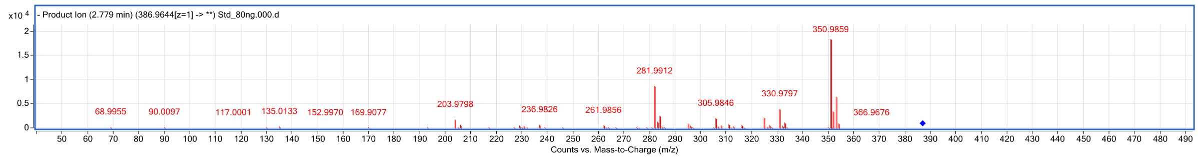

Fipronil sulfone (20 eV)

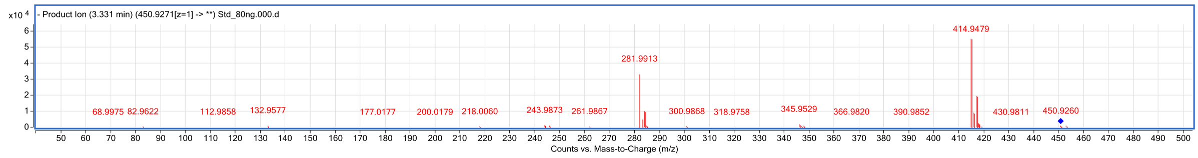

S2 Fig. High-resolution electrospray ionisation tandem mass spectrometry (HR-ESI-MS/MS) fragmentation of fipronil, fipronil desulfinyl and fipronil sulfone.
