# Supplementary figures and images for: Non-detection of honeybee hive contamination following Vespula wasp baiting with protein containing fipronil
Source: PLoS One. 2018 Oct 29;13(10):e0206385. doi: 10.1371/journal.pone.0206385 (PMC6205613; doi:10.1371/journal.pone.0206385)

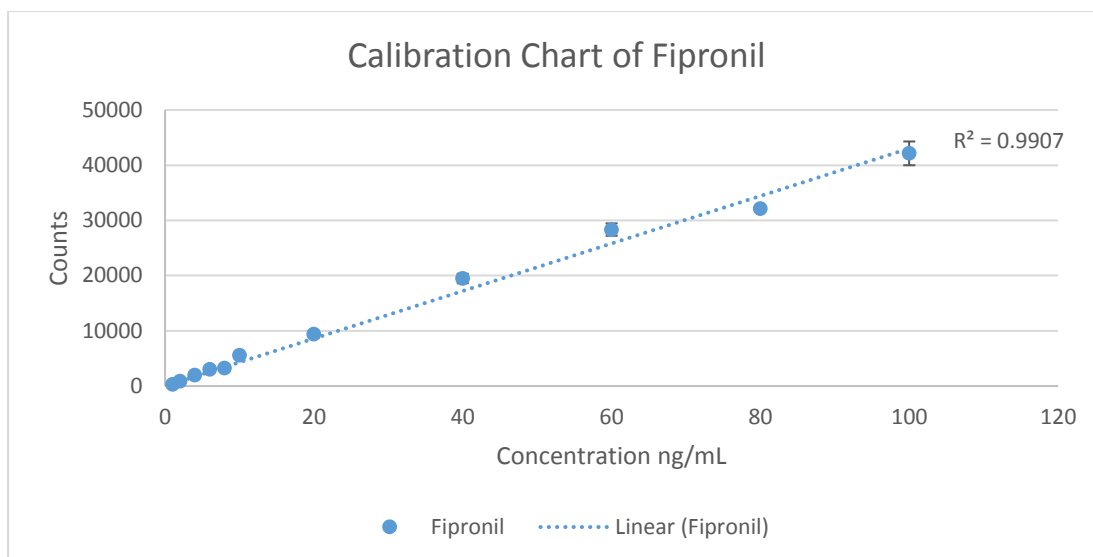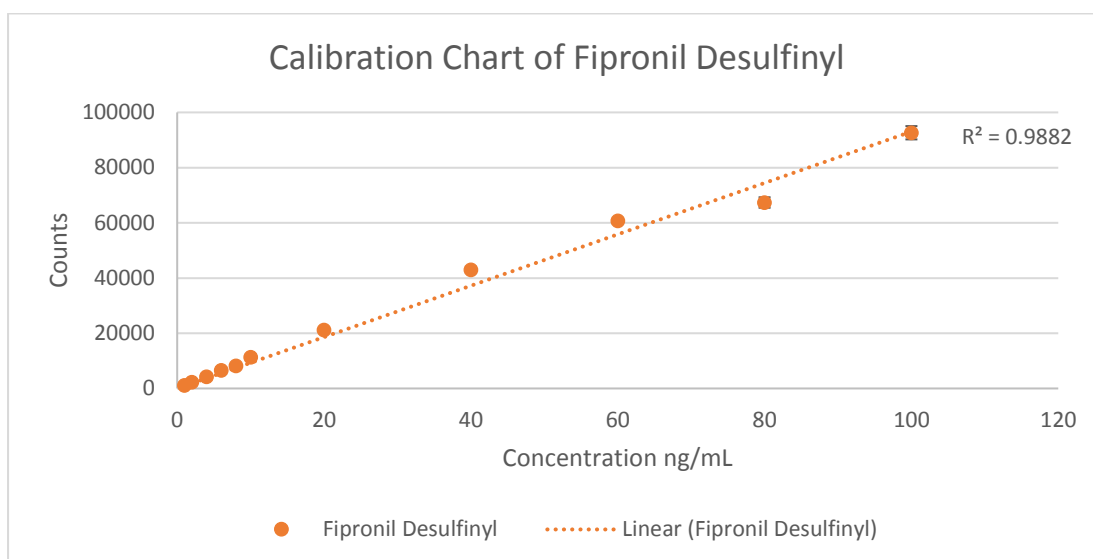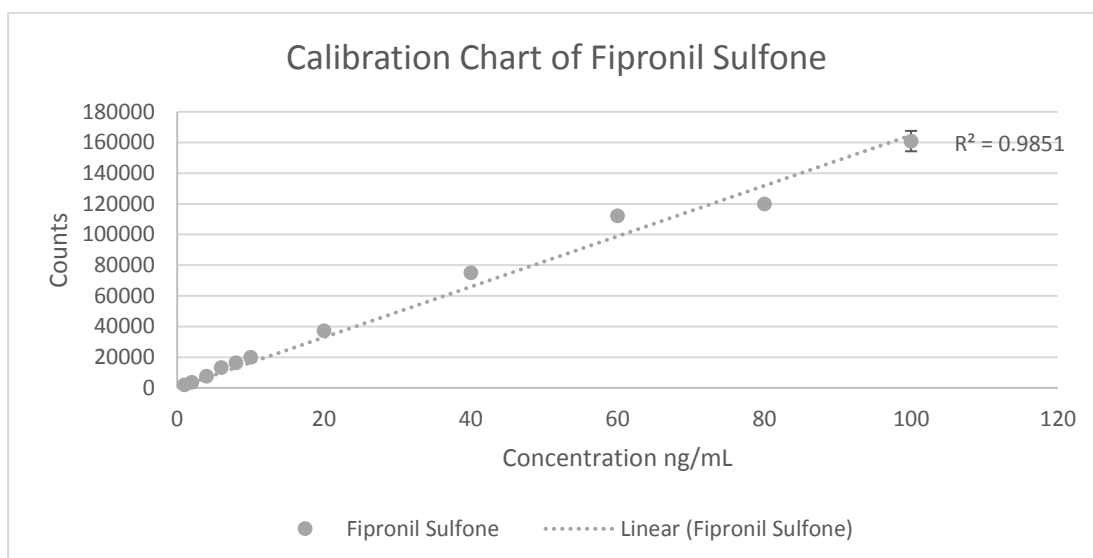

**S3 Fig. Calibration curves for fipronil and its derivatives. Values are means  $\pm$  standard errors.**

Supplement: S3 Fig — Values are means ± standard errors. (PDF) [file pone.0206385.s003.pdf]
