# Supplementary material for: Non-detection of honeybee hive contamination following Vespula wasp baiting with protein containing fipronil
Source: PLoS One. 2018 Oct 29;13(10):e0206385. doi: 10.1371/journal.pone.0206385 (PMC6205613; doi:10.1371/journal.pone.0206385)
Supplement: S1 Table — (PDF) [file pone.0206385.s004.pdf]

S1 Table. Calculations of figures of merit for fipronil and its derivatives.

Fipronil

ng/mL	QuEChERS treatment	No treatment	Recovery %	Difference from average recovery	Difference squared
100	42150	132188	31.9%	−3.8%	0.14%
80	32130	101574	31.6%	−4.0%	0.16%
60	28344	76012	37.3%	1.6%	0.03%
40	19487	51708	37.7%	2.0%	0.04%
20	9400	24894	37.8%	2.1%	0.04%
10	5618	13761	40.8%	5.2%	0.27%
8	3260	10009	32.6%	−3.1%	0.09%
6	3059	7928	38.6%	2.9%	0.09%
4	1994	5258	37.9%	2.3%	0.05%
2	914	3048	30.0%	−5.7%	0.32%
1	342	951	36.0%	0.3%	0.00%

Fipronil	Average recovery	35.6%	%RSD	11.1%

Fipronil desulfinyl

ng/mL	QuEChERS treatment	No treatment	Recovery %	Difference from average recovery	Difference squared
100	92626	247805	37.4%	−7.4%	0.54%
80	67366	194182	34.7%	−10.0%	1.01%
60	60649	143039	42.4%	−2.3%	0.05%
40	43012	93425	46.0%	1.3%	0.02%
20	21148	48547	43.6%	−1.2%	0.01%
10	11321	23447	48.3%	3.5%	0.13%
8	8164	18684	43.7%	−1.0%	0.01%
6	6569	13391	49.1%	4.3%	0.19%
4	4237	9303	45.5%	0.8%	0.01%
2	2287	5117	44.7%	0.0%	0.00%
1	1126	1985	56.7%	12.0%	1.43%

Fipronil desulfinyl	Average recovery	44.73%	%RSD	5.56%

Fipronil sulfone

ng/mL	QuEChERS treatment	No treatment	Recovery %	Difference from average recovery	Difference squared
100	160915	535216	30.1%	−7.3%	0.53%
80	119902	411878	29.1%	−8.3%	0.68%
60	112164	300531	37.3%	0.0%	0.00%
40	75080	203191	37.0%	−0.4%	0.00%
20	37165	100793	36.9%	−0.5%	0.00%
10	19902	53348	37.3%	−0.1%	0.00%
8	16470	39066	42.2%	4.8%	0.23%
6	13155	29074	45.2%	7.9%	0.62%
4	7688	19099	40.3%	2.9%	0.08%
2	3590	10268	35.0%	−2.4%	0.06%
1	1917	4707	40.7%	3.4%	0.11%

Fipronil sulfone	Average recovery	37.36%	%RSD	4.60%
