# Supplementary material for: Non-detection of honeybee hive contamination following Vespula wasp baiting with protein containing fipronil
Source: PLoS One. 2018 Oct 29;13(10):e0206385. doi: 10.1371/journal.pone.0206385 (PMC6205613; doi:10.1371/journal.pone.0206385)
Supplement: S2 Table — (PDF) [file pone.0206385.s005.pdf]

S2 Table. Summary of published fipronil LD_{50} toxicity data for honeybees (*Apis mellifera*).

Primary source	Fipronil/derivative	Oral/dermal	Value reported	Converted ~ng/bee	Timeframe	Notes
Rhône-Poulenc [1]	Fipronil	Oral	0.004 mg/bee	4,000 ng	?	Cited in Tingle et al. [14] as LD_{50} 0.004 mg/bee [1]. May have meant some unit other than milligram, e.g. µg/bee.
Mayer & Lunden [2]	Fipronil	Dermal	0.013 µg/bee	13	24 h	
Decourtye [3]	Fipronil	Oral	6 ng/bee	6	48 h	Cited in Decourtye et al. [15].
El Hassani et al. [4]	Fipronil	Dermal	<5 ng/harnessed bee	<5	?	The fipronil was dissolved in acetone and then diluted. The final concentration of acetone in sucrose and watery solutions was equal to 0.1% (vol./vol.).
European Food Safety Authority (EFSA) [5]	Fipronil	Oral	0.00417 µg/bee	4	?	See page 98.
Kievits & Bruneau [6]	Fipronil	Oral	4.17 ng/bee	4	?	
Kievits & Bruneau [6]	Fipronil	Dermal	5.93 ng/bee	6	?	
Li et al. [7]	Fipronil (range encompasses: R(-)-enantiomer, S(+)-enantiomer & racemate)	Dermal	3.45–3.86 ng/bee	4	48 h	No significant difference in the varieties of fipronil on the LD_{50} figure. Anesthetised with ethyl ether and dose applied in an acetone solution – no control group mortality reported.
Li et al. [7]	Fipronil (range encompasses: R(-)-enantiomer, S(+)-enantiomer & racemate)	Oral	0.037–0.053 mg/L	7	48 h	Bees were fed *ad libitum* with fipronil in an acetone and sucrose solution – no control group mortality reported. No significant difference in the varieties of fipronil on the LD_{50} figure.
Li et al. [7]	Fipronil (range encompasses: R(-)-enantiomer, S(+)-enantiomer & racemate)	Dermal	12.0–13.4 ng/bee	13	24 h	No significant difference in the varieties of fipronil on the LD_{50} figure. Anesthetised with ethyl ether and dose applied in an acetone solution – no control group mortality reported.
Li et al. [7]	Fipronil (range encompasses: R(-)-enantiomer, S(+)-enantiomer & racemate)	Oral	0.037–0.053 mg/L	7	24 h	Bees were fed *ad libitum* with fipronil in an acetone and sucrose solution – no control group mortality reported. No significant difference in the varieties of fipronil on the LD_{50} figure.
Carrillo et al. [8]	Fipronil	Oral	0.28 µg/bee	280	?	As in many other oral dose studies, LD_{50} calculated from an average response to group consumption of a lethal concentration.
Roat et al. [9]	Fipronil	Dermal	1.06 ng/bee	1	24 h	Noted colonies tested were newly emerged Africanised honeybees that originated from a cross between the European subspecies *A. mellifera mellifera* and the African subspecies *A. m. scutellata*.
Roat et al. [9]	Fipronil	Oral	1.27 ng/bee	1	24 h	
Carvalho et al. [10]	Fipronil	Dermal	5.83 ng/bee	6	48 h	Value apparently corrected for the associated acetone control and a second control with no acetone. Mortality from these apparently ≤5% but no mortality metrics reported.
Zaluski et al. [11]	Fipronil	Oral	0.19 µg/bee	190	24 h	Authors report "A solution of 1 g L^{-1} Regent 800WG was prepared in distilled water, and the doses were diluted from this solution". This is in contrast with other studies above where acetone was used and

						residual trace acetone may have been present.
Zaluski et al. [11]	Fipronil	Dermal	0.009 μg/bee	9	24 h	
Lunardi et al. [12]	Fipronil	Oral	0.0528 μg/bee	53	24 h	As in Zaluski et al. [11], no acetone was used in the tests. The tests involved bees from a cross between the European subspecies *A. mellifera mellifera* and the African subspecies *A. m. scutellata.*
Lunardi et al. [12]	Fipronil	Dermal	0.0054 μg/bee	5	24 h	
Bovi et al. [13]	Fipronil	Oral	0.2316 μg/bee	232	24 h	No acetone was used in the tests. The tests involved the African subspecies *A. mellifera scutellata.*
Bovi et al. [13]	Fipronil	Dermal	0.0080 μg/bee	8	24 h	
European Food Safety Authority (EFSA) [5]	Fipronil sulfone	Oral	0.0064 μg/bee	6	?	Fipronil sulfone coded as MB 46136 in this report.
None	Fipronil desulfinyl	Oral/dermal	-	-		No published study for *A. mellifera* LD_{50} found.

S1 Table references

1. Rhône-Poulenc. Atelier International Fipronil/lutte antiacridienne. Lyon, May 3–5 1995. Unpublished report. Lyon: Rhône-Poulenc Agrochimie; 1995.
2. Mayer DF, Lunden JD. Field and laboratory tests of the effects of fipronil on adult female *Apis mellifera*, *Megachile rotundata* and *Nomia melanderi*. J Apic Res. 1999; 38:191–7.
3. Decourtye A. Etude de l’impact de produits phytopharmaceutiques sur la survie et l’apprentissage associatif chez l’abeille domestique (Apis mellifera L.). PhD Thesis, University of Paris-Sud, Orsay, France. 2002. Available from http://www.theses.fr/2002PA112111.
4. El Hassani AK, Dacher M, Gauthier M, Armengaud C. Effects of sublethal doses of fipronil on the behavior of the honeybee (*Apis mellifera*). Pharmacol Biochem Behav. 2005; 82:30–9.
5. European Food Safety Authority. Conclusion regarding the peer review of the pesticide risk assessment of the active substance fipronil. EFSA J. 2006; 65:1–110.
6. Kievits J, Bruneau E. Neurotoxiques systémiques, un risque pour les abeilles? Abeilles & Cie. 2007; 118:12–7.

7. Li X, Bao C, Yang D, Zheng M, Li X, Tao S. Toxicities of fipronil enantiomers to the honeybee *Apis mellifera* L. and enantiomeric compositions of fipronil in honey plant flowers. Environ Toxicol Chem. 2010; 29(1):127–32.
8. Carrillo MP, Bovi TdeS, Negrão AF, Orsi RdeO. Influence of agrochemicals fipronil and imidacloprid on the learning behavior of *Apis mellifera* L. honeybees. Acta Sci Anim Sci. 2013; 35(4):431–4.
9. Roat TC, Carvalho SM, Nocelli RCF, Silva-Zacarin ECM, Palma MS, Malaspina O. Effects of sublethal dose of fipronil on neuron metabolic activity of Africanized honeybees. Arch Environ Contam Toxicol. 2013; 64:456–66.
10. Carvalho SM, Belzunces LP, Carvalho GA, Brunet J-L, Badiou-Beneteau A. Enzymatic biomarkers as tools to assess environmental quality: a case study of exposure of the honeybee *Apis mellifera* to insecticides. Environ Toxicol Chem. 2013; 32(9):2117–24.
11. Zaluski R, Kadri SM, Alonso DP, Ribolla PEM, de Oliveira Orsi R. Fipronil promotes motor and behavioural changes in honey bees (*Apis mellifera*) and affects the development of colonies exposed to sublethal doses. Environ Toxicol Chem. 2015; 34:1062–9.
12. Lunardi JS, Zaluski R, Orsi RO. Evaluation of motor changes and toxicity of insecticides fipronil and imidacloprid in Africanized honey bees (Hymenoptera: Apidae). Sociobiology. 2017; 64(1):50–6.
13. Bovi TS, Zaluski R, Orsi RO. Toxicity and motor changes in Africanized honey bees (*Apis mellifera* L.) exposed to fipronil and imidacloprid. An Acad Bras Ciênc. 2018; 90(1):239–45.
14. Tingle CCD, Rother JA, Dewhurst CF, Lauer S, King, WJ. Health and environmental effects of fipronil. Pesticide Action Network UK briefing AI1; 2000.
15. Decourtye A, Devillers J, Genecque E, Le Menach K, Budzinski H, Cluzeau S, et al. Comparative sublethal toxicity of nine pesticides on olfactory learning performances of the honeybee *Apis mellifera*. Arch Environ Contam Toxicol. 2005; 48:242–50.
